# Neuroimaging Techniques as Descriptive and Diagnostic Tools for Infants at Risk for Autism Spectrum Disorder: A Systematic Review

**DOI:** 10.3390/brainsci12050602

**Published:** 2022-05-05

**Authors:** Maria J. Ayoub, Laura Keegan, Helen Tager-Flusberg, Simone V. Gill

**Affiliations:** 1 College of Health and Rehabilitation Sciences: Sargent College, Boston University, 635 Commonwealth Avenue, Boston, MA 02215, USA; mjayoub@bu.edu (M.J.A.); lakeegan@bu.edu (L.K.); 2 Department of Psychological and Brain Sciences, Boston University, Boston, MA 02215, USA; htagerf@bu.edu

**Keywords:** autism spectrum disorder, neuroimaging

## Abstract

Autism Spectrum Disorder (ASD) has traditionally been evaluated and diagnosed via behavioral assessments. However, increasing research suggests that neuroimaging as early as infancy can reliably identify structural and functional differences between autistic and non-autistic brains. The current review provides a systematic overview of imaging approaches used to identify differences between infants at familial risk and without risk and predictive biomarkers. Two primary themes emerged after reviewing the literature: (1) neuroimaging methods can be used to describe structural and functional differences between infants at risk and infants not at risk for ASD (*descriptive*), and (2) neuroimaging approaches can be used to predict ASD diagnosis among high-risk infants and developmental outcomes beyond infancy (*predicting later diagnosis*). Combined, the articles highlighted that several neuroimaging studies have identified a variety of neuroanatomical and neurological differences between infants at high and low risk for ASD, and among those who later receive an ASD diagnosis. Incorporating neuroimaging into ASD evaluations alongside traditional behavioral assessments can provide individuals with earlier diagnosis and earlier access to supportive resources.

## 1. Introduction

In the U.S., it is estimated that 1 in every 44 children are diagnosed with Autism Spectrum Disorder (ASD) [1]. Core diagnostic criteria for ASD include the presence of restrictive, repetitive behaviors (RRBs) and differences in communication and social interactions [2]. RRBs and limits in communication and social interactions are often accompanied by autistic children’s developmental delays in motor [3,4], language, and communication skills [5]. Early, comprehensive, and accurate diagnostic assessments are crucial in meeting each autistic individual’s support needs across the lifespan and enhancing their overall quality of life.

ASD diagnosis relies on a behavioral phenotype (i.e., RRBs and impaired communication and social skills) and, at the earliest, can be diagnosed in the second year of life [2,6]. Yet, many children with ASD receive diagnosis after the second year of life [6]. Many children are also undiagnosed during an initial evaluation despite having sufficient scores to warrant a diagnosis, and some diagnoses are delayed simply because some children do not meet the cut-off score until they are re-evaluated at an older age [7]. Many individuals, females in particular, may exhibit different behavioral phenotypes than males, and are more likely to mask their behaviors to socially conform [8]. Belonging to an ethnic minority group is also associated with receiving a late diagnosis [7]. This can lead to years of delayed diagnosis and, as a result, prolonged, unmet support needs.

Despite the dependence on a behavioral phenotype to diagnose ASD, ASD is a biologically based neurodevelopmental disorder resulting from altered brain development that begins early in ontogeny [9]. Thus, assessment and diagnosis strongly benefit from additional types of non-behavioral assessments. An aspiration is to be able to supplement behavior with neural measures to reduce the risk of delayed or missed diagnosis [7].

The past decade has seen growth in studies investigating early brain development in infants prior to diagnosis using different types of technology: structural brain measures using magnetic resonance imaging (MRI) and diffusor tensor imaging (DTI), and functional measures derived from functional magnetic resonance imaging (fMRI), electroencephalography (EEG), and functional near-infrared spectroscopy (fNIRS).

Recent work has investigated using brain imaging to identify neurological biomarkers of ASD in infancy [10], which would pose an advantage over behavioral phenotyping for diagnosing ASD for several reasons. First, behavioral signs of ASD have been documented during the first two years of life but have not been adequate in predicting later ASD diagnosis [11]. Second, machine learning algorithms have projected results from infant brain imaging to be more cost effective than other diagnostic approaches for identifying ASD [12]. Implementing neural measures to identify risk and to predict later diagnosis may serve as more robust screening tools than those currently available.

We conducted a systematic review to investigate how brain imaging studies have been used in the infancy period and what they have found. Previous narrative reviews have provided helpful information on brain biomarkers that could be used to screen for ASD in infancy. The current paper adds to the existing literature by providing a systematic investigation of imaging approaches used to identify differences between infants at familial risk and without risk and predictive biomarkers.

## 2. Methods

### 2.1. Study Design

A comprehensive systematic review was conducted to pinpoint differences between infants at familial risk and without risk as well as predictive biomarkers. The methodology followed the guidelines specified in the Preferred Reporting Items for Systematic Reviews and Meta-Analyses (PRISMA) checklist [13].

### 2.2. Search Strategy

The authors worked with the Boston University Sargent College of Health and Rehabilitation Sciences medical librarian to develop and execute a literature search. Four electronic databases were deemed appropriate for the research topic: PubMed, Embase, PsycInfo, and Cumulative Index of Nursing and Allied Health Literature (CINAHL). Search terms were developed to include the population demographics and means of assessment (Table 1). Search results were exported to Mendeley, a reference management software, and shared among the authors for review and selection based on inclusion and exclusion criteria. This search was supplemented with references on the Baby Siblings Research Consortium (BSRC) website: https://www.babysiblingsresearchconsortium.org/publications (accessed on 12 January 2022).

### 2.3. Inclusion and Exclusion Criteria

Studies were included if they were written in English, peer-reviewed, focused on infants (birth – 3 years of age) genetically predisposed to autism, and included neuroimaging techniques. Studies were excluded if they were focused on populations with neurodevelopmental disorders other than autism, included preterm or low birth weight infants, did not include neuroimaging methods, or used neuroimaging for purposes other than describing and diagnosing these individuals.

### 2.4. Data Extraction

**Figure 1:**
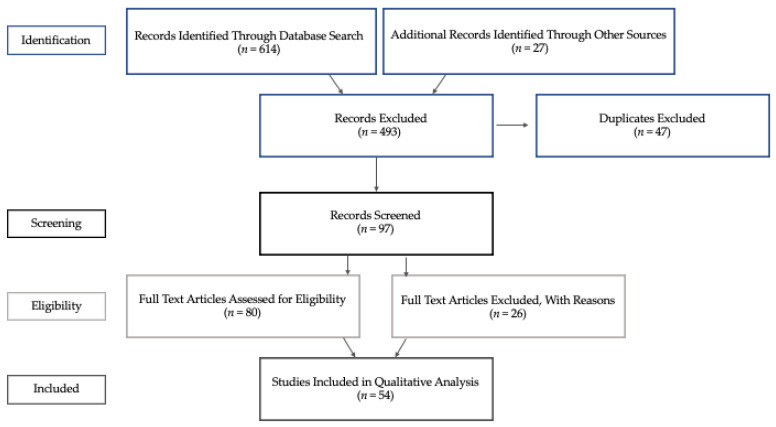
Breakdown of data extraction.

The authors conducted a preliminary screen of the article titles and abstracts based on the research question and inclusion/exclusion criteria. Full-text articles were gathered and then reviewed by the authors. Articles were included if they were relevant to the research question and fit the eligibility criteria (Figure 1). An evidence table was created in order to summarize levels of evidence, participant characteristics, study design, and methods (Table S1).

## 3. Results

### 3.1. Study Selection

Using the search strategy mentioned, 614 articles were found. The articles covered a wide range of types of neuroimaging, including fNIRS, MRI, fMRI, and electroencephalography (EEG). Two primary themes emerged after fully reading the articles that we determined fit our inclusion criteria (*n* = 54): (1) using neuroimaging to describe structural and functional differences between infants at risk and infants not at risk for ASD (*descriptive*), and (2) using neuroimaging to predict ASD diagnosis among high-risk infants and developmental outcomes beyond infancy (*diagnostic*).

### 3.2. Descriptive Articles

Twenty-five articles were classified as descriptive. The majority of these articles (*n* = 16) were cross-sectional studies comparing low-risk (LR) and high-risk (HR) infants within their first year of life (before a diagnostically viable age) or within their first 2 years of life before receiving diagnosis. Infants who were considered HR for ASD were defined as having at least one sibling diagnosed with ASD and LR infants had no familial history of ASD diagnosis. Longitudinal studies that were descriptive (*n* = 9) either compared diagnosed autistic children to non-autistic counterparts or HR without confirmed diagnosis versus LR at multiple timepoints across the first two years of life. Two of these longitudinal studies explored a much wider developmental trajectory—children (age 1.5–5 years) who showed ASD symptoms at 48 months [14] and a much broader age range from 3 to 42 years old [15]. These studies used several types of neuroimaging techniques to describe structural and functional differences between LR and HR groups: fNIRS (*n* = 4), fMRI (*n* = 2), MRI (*n* = 5), and EEG (*n* = 14).

#### 3.2.1. Structural Findings

Compared to LR infants, HR infants have decreased T1-weighted/T2-weighted (T1w/T2w) ratio: an indirect measure of myelination obtained from MRI scans [16]. Enlarged grey and white matter in the temporal, frontal, and cingulate cortices were also observed in HR infants [14]. However, there were no differences in radiological readings, head circumference, or MRI volumetric measures between HR and LR infants [17] and no increases in cerebrospinal fluid after 4 years of age into adulthood in HR infants [15].

#### 3.2.2. Functional Findings

Several articles highlighted differences between HR and LR infants regarding regional activation and response to stimuli, generally finding that HR infants exhibit either reduced, atypical, or nonsignificant neurological responses compared to their LR counterparts.

##### Resting fMRI/EEG

Findings regarding changes in connectivity among HR infants vary. Resting fMRI has shown that children with ASD exhibit atypical functional connectivity differences in a variety of resting-state networks. For example, HR infants experience an overall lack of changes in connectivity in language-related regions within the first year of life [18]. Additionally, differences in power of resting EEG have been associated with cognitive functions that are associated with ASD, such as language and temperament, and may have implications for differentiating between HR and LR infants [19]. Regarding spectral power, HR infants exhibit lower spectral power in frontal regions across delta, theta, low alpha, high alpha, beta, and gamma frequency bands compared to LR infants at 6 months of age [19]. Beyond this age and within the first two years of life, HR infants display a different developmental trajectory in changes in spectral power compared to their LR counterparts [19]. Relationships between frontal power and sensory responsiveness in HR infants also exist; one study found that lower levels of sensory responsiveness were associated with higher alpha band synchronization in occipital and temporal regions and higher theta connectivity between frontal and posterior regions [20].

##### Visual and Social Stimuli

Relative to LR infants, HR infants show reduced cortical responses to social stimuli in the posterior temporal cortex [21,22] and bilateral fronto-parieto-temporal cortices [23]. Several studies have examined neural responses to faces among HR and LR infants, with varied results. For example, HR infants with more positive affect display a more distinguished P400 response to gaze stimuli, with faster responses to faces looking towards the infant [24]. Research has also shown no significant differences between LR and HR groups in neural responses to faces [25,26]. However, there is a developmental lag in negative central (Nc) responses among the HR group, which holds implications for attention allocation [25]. Other studies in which infants were shown familiar and novel faces revealed that both HR and LR infants are able to differentiate between the two, as indicated by appropriate variations in N290 and P400 amplitudes and Nc response [27]. Interestingly, only LR infants showed a delayed response to the novel faces, as indicated by P400 latency. Shorter Nc latency to the familiar face was associated with stronger interpersonal skills across both HR and LR infants [27]. When differentiating between the two types of faces, researchers also found that LR and HR infants used different brain mechanisms to do so—changes in facial features were associated with fluctuations in N290, an indicator of facial perception, only among LR infants [28]. LR and HR infants also process positive facial affect differently—in response to subtle facial expressions, LR infants exhibit significantly longer processing (P400 latency) and increased attention (Nc amplitude) [29].

HR infants also show atypical responses to different types of visual and social stimuli; for example, HR infants react more quickly to objects and more slowly to faces than LR infants as shown by latencies of N290 and P400 ERP components [30]. Other research has shown that while both LR and HR infants demonstrate increased N290 amplitudes in response to faces rather than toys, the HR group exhibits smaller N290 amplitudes overall, as well as significantly smaller Nc responses [31]. Interestingly, one study examining ERP responses to familiar and unfamiliar faces and objects found that compared to LR infants, HR infants demonstrate an atypical lack of hemispheric asymmetry [30]. Early intervention research indicates that interventions addressing social attention between 9 and 11 months of age provides the opportunity for HR infants to develop ERP responses to faces versus objects that are similar to the responses found in LR infants [32].

##### Auditory and Language Stimuli

Differences in activation were also found for auditory stimuli. Compared to LR infants, HR infants exhibit less activation in response to voice versus non-voice sounds in the middle and superior temporal areas and the medial frontal gyrus [33], to human voices in the right posterior temporal cortex [21], to sad vocalizations in the right fusiform gyrus and left hippocampus [33], and to voice stimuli in the right temporal and medial frontal regions reflecting a lack of early specialization for voice processing [33]. HR female infants also do not exhibit a habituation response to repetitive auditory stimuli in the right and left temporal regions. LR counterparts showed decreased activation in these areas over the duration of the exposure, but HR females did not express changes in activation levels [34]. Compared to LR infants, HR infants demonstrate ERPs with higher amplitudes in response to repetitive consonant-vowel stimuli; furthermore, there is a positive correlation between these amplitudes and language ability [35]. LR infants between the age of 6 and 12 months demonstrate a lateralization response to speech sounds, while HR infants do not [36]. Along with differing levels of activation, HR infants also show other patterns of symmetry and lateralization compared to LR infants. For example, 6-month old HR infants have different patterns in alpha asymmetry than LR infants; HR infants show left relative frontal asymmetry, but LR infants show right relative frontal asymmetry [37], which may lead to differences in brain development growth trajectories between HR and LR infants [37]. Intra-hemispheric connectivity, particularly left anterior-left posterior connectivity, also decreased over time in HR infants between 3 and 12 months of age in response to trisyllabic sequences [38].

### 3.3. Articles Predicting Later Diagnosis

Twenty-nine articles were classified as predicting later diagnosis, and nearly all of them (*n* = 27) examined longitudinal data. The majority of the articles (*n* = 27) compared LR and HR infants in infancy and compared them again once the presence of ASD symptomatology and/or an ASD diagnosis (or lack thereof) were confirmed among HR infants. Outcomes were generally assessed at 24 or 36 months using standard behavioral assessments and are usually combined with clinical judgment. One study included only HR infants, and two studies included infants that developed a language delay or displayed atypical development other than ASD (n = 2). These studies used several types of neuroimaging to describe structural and functional differences between LR and HR groups: EEG (*n* = 13), MRI (*n* = 13, 5 of which involved DTI), fNIRS (*n* = 2), and fMRI (*n* = 1).

#### 3.3.1. Structural Findings

A variety of structural findings have been identified in HR infants that proceed to receive an ASD diagnosis (HR+). Compared to HR infants who do not receive a diagnosis (HR negative, or HR-) and LR infants, HR+ infants have decreased grey matter and increased white matter [39]. HR+ infants also exhibit increased fractional anisotropic (FA) levels at 6 months of age, with slowed developmental change over time relative to their LR counterparts, resulting in decreased FA in HR+ infants at 24 months of age [40].

HR+ infants also show increased total brain volume (TBV) growth rates in their second year of life compared to HR- and LR infants. Moreover, increased TBV is associated with increased ASD symptom severity [41]. Increased cerebrospinal fluid (CSF) is also associated with ASD symptom severity, and HR+ infants have more CSF than LR infants [42]. HR+ infants have increased extra-axial fluid [43], increased cerebral volume [43], and increased area and thickness of the corpus callosum in the first year of life, which are correlated with RRBs in the second year of life [44]. Increased cerebellar and subcortical volumes in 4- to 6-month old HR+ infants are associated with RRBs at 36 months old [45].

Differences in more specific brain regions have also been observed. Difficulties with sleep onset are related to hippocampal volume trajectories in HR+ infants [46]. HR groups show no change in activation levels across all ROIs in response to speech at 6 months old, which predict lower verbal developmental quotient (VDQ) at 24 months old [47]. In contrast, LR infants exhibit a greater response in left anterior ROIs, which predict VDQ scores. Although visual orientation latencies were longer in HR+ infants, there was no association between these latencies and the microstructure of the splenium of the corpus callosum—this association was only found in LR infants [48]. As early as 6 weeks, HR infants show altered structural connectivity in the dorsal language network and higher fractional anisotropy than LR infants in the right superior longitudinal fasciculus (SLF), as well as more right lateralization of the SLF than LR infants [49]. Both lateralization and fractional anisotropy levels are associated with higher social communication impairments at 18 months as demonstrated with higher ADOS scores [49]. Network efficiency was associated with increased ASD symptom severity [50] In 6-month old HR infants, lower network inefficiencies in auditory processing and language areas (i.e., Broca’s and Wiernicke’s areas) were predictive of future autism symptom severity in behavioral, motor, visual, and somatosensory domains demonstrated with the ADOS at 24 months [50].

#### 3.3.2. Functional Findings

Expanding upon the general consensus of the descriptive articles, several diagnostic articles showed that HR infants who went on to receive an ASD diagnosis (HR positive, or HR+), exhibit differences in both resting data and responses to stimuli compared to their LR counterparts.

##### Resting EEG/fMRI

HR infants display different levels of connectivity compared to LR infants. HR infants exhibit low levels of connectivity in thalamo-prefrontal, thalamo-occipital, and thalamo-motor areas compared to their LR counterparts [51]. Atypical connectivity predicts atypical social development at 9 and 36 months of age [51], and higher temporoparietal connectivity and lower frontal connectivity at 3 months predicts increased ASD symptomatology at 18 months [52]. Additionally, patterns of functional connectivity are associated with RRBs in HR+ infants [53,54].

Studies have shown that compared to LR infants, HR infants exhibit reduced frontal high alpha and beta power at 3 months of age; although this did not predict diagnostic outcome, reduced high alpha power was later associated with poor expressive language at 12 months [55]. Another study found that HR toddlers exhibit reduced frontal gamma power compared to LR toddlers, which was associated with better expressive language abilities among both HR- and HR+ toddlers [56]. Furthermore, the relationship between EEG measures and language are dependent upon risk status [57]. Outcomes other than language have also been studied—compared to LR infants, HR infants express resting frontal asymmetry, which was associated with increased sensory seeking. Sensory seeking was predictive of later social symptomology [58].

##### Visual and Social Stimuli

Relative to LR infants, HR+ infants show decreased activation to visual social stimuli across several areas, including inferior frontal and posterior temporal regions [59]. In response to being shown familiar and unfamiliar faces, HR infants (particularly HR+ infants) displayed increased leftward lateralization compared to their LR counterparts, who exhibited rightward lateralization [60]. HR infants at 6 months who were later determined HR+ at 24 months exhibited faster P400 and shorter and smaller (i.e., less sustained) Nc responses to faces [61]. In response to watching videos, HR+ infants exhibited increased phase-lagged alpha-range connectivity compared to their HR- and LR counterparts; additionally, there was a strong relationship between hyperconnectivity at 14 months and severity of RRB’s at 3 years in HR+ infants [62].

##### Auditory and Language Stimuli

HR+ infants exhibit decreased activation to vocal sounds and increased activation to non-vocal sounds in left lateralized temporal regions [59]. Twenty-four-month-old HR+ infants also display decreased activation (i.e., decreased oxygenated hemoglobin concentration) across bilateral anterior regions of interest in response to hearing random and repeated syllable sequences [63]. Similar to the results for a descriptive study finding that HR infants express a lack of neural habituation to repetitive auditory stimuli [34], one diagnostic study showed that HR+ infants exhibit decreased suppression of cortical activity to repetitive tones [64]. Another study comparing neural responses to “early” words (i.e., words that one would expect an infant to acquire by 18 months) and “late” words showed that, compared to LR and HR+ infants, the HR group with some ASD symptoms exhibited atypical responses to late words in the temporo-parietal regions [65]. The study also found a relationship between ASD severity and neural responses to late words in the left frontal regions [65]. In response to hearing speech sounds, HR+ infants at 12 months exhibited reduced functional connectivity compared to HR- and LR infants [66]. Furthermore, compared to infants without a diagnosis, HR+ infants at 12 months exhibited reversed lateralization in response to speech sounds [67].

## 4. Discussion

The purpose of this systematic review was to comprehensively assess the neuroimaging techniques and subsequent structural and functional findings that have been used to both identify and later diagnose infants at risk for ASD. We found that several types of neuroimaging, including fNIRS, fMRI, MRI, and EEG, have been used to identify structural and functional differences between infants at high and low risk for ASD.

Observations of brain differences are most useful if they are associated with later ASD diagnoses and can be used for early detection of ASD. Studies that focus on using imaging techniques to predict later ASD diagnoses separated infants into HR infants who were later diagnosed, HR infants who did not go on to be diagnosed, and LR infants. HR infants who went on to be diagnosed with ASD had less grey matter and more white matter [39], larger cerebral volume [43,45], faster brain volume growth rates [41], and more cerebrospinal [42] and extra-axial fluid [43]. Structural differences were related to specific ASD symptoms such as RRBs; larger volume in cerebellar and subcortical regions along with thicker corpus callosum measures were associated with more RRBs [44,45].

Findings on brain activation in HR and LR infants confirmed that differences between the groups extend beyond structural divergences. Of note, lower activation for HR versus LR infants already highlights hallmark signs of ASD in infancy; HR infants have less activation to voice sounds [33] and sad vocalizations, and do not habituate to repetitive auditory stimuli [34]. Results on lower activation in HR infants in other brain areas also relate to ASD signs such as less activation in the parietal cortex to social stimuli and stronger neural responses to objects compared to faces.

Similar to findings from descriptive studies, there were differences in activation for HR ASD infants later diagnosed with ASD compared to HR not diagnosed and LR infants. Those later diagnosed showed decreased activation to visual social stimuli (interior frontal and posterior temporal area) and decreased activation to vocal sounds and increased activation to nonvocal sounds in left temporal regions [59]. They also demonstrated decreased activation to syllable sequences in bilateral anterior regions of interest [63]. Responses to different types of stimuli and habituation responses are crucial factors in allocating cognitive resources to relevant stimuli in the environment. When these neural responses are hindered, the ability to process and attend to more complex stimuli that are part of daily life may be limited [34]. Limited activation and lack of habituation are also associated with reduced receptive language skills and social skills in autistic children, making these early neural responses a potential biomarker for developmental trajectories across language and social domains [64].

Ultimately, neuroimaging is a sophisticated, comprehensive, and supportive addition to the assessment and diagnosis of ASD. Neuroimaging studies in HR infants and those who proceed to receive an ASD diagnosis have established that there are, indeed, consistent and notable neuroanatomical and neurological findings within this population. Importantly, these differences are identifiable much earlier in life than those identified via traditional behavioral assessments. Including neuroimaging alongside behavioral assessments would strongly benefit and expedite the diagnostic process, paving the way to promote accessibility and meet individuals’ support needs as early in life as possible. In addition to early diagnosis, neuroimaging can help health practitioners and caregivers better understand children’s developmental course in related domains, such as language [47,49] and motor development [68,69]. An earlier and comprehensive understanding of individual differences may allow caregivers to support children’s developmental trajectories and access services that improve quality of life across the lifespan.

### Limitations

Our paper has several limitations. First, although systematic review protocols are well-defined, relevant articles may be omitted due to constraints imposed by the protocol. Second, publication bias may cause important findings to be left out; studies that do not demonstrate statistical significance can lead systematic reviews to be biased towards only including studies with statistically significant results. Last, much work still needs to be done to create personalized interventions with the current research knowledge available, to predict ASD diagnosis on an individual level, and to translate the findings for use in clinical settings.

## 5. Conclusions

Including neuroimaging techniques in the early assessment and diagnosis of ASD is strongly supported by the current literature. Neuroimaging affords the opportunity to objectively and reliably identify structural and functional brain differences in HR infants earlier than traditional behavioral assessments. These neuroimaging findings can be used to predict ASD diagnosis and other related developmental outcomes and ultimately provide individuals with early diagnosis, earlier and more robust access to their support needs, and enhanced quality of life.

## Figures and Tables

**Table 1:** Search Terms.

Search line 1	Search line 2	Search line 3	Search line 4
autism spectrum disorder [MeSH] autis *	infant[MeSH] infan * baby babies newborn	risk[MeSH] risk genetic predisposition[MeSH] disease susceptibility[MeSH] predispos * genetic genetic predisposition disease susceptibility	diagnostic imaging[MeSH] neuroimaging[MeSH] diagnostic imaging neuroimaging brain imaging brain scintiscanning magnetic resonance imaging nuclear magnetic resonance imaging mri

* Used at the end of root of search term for expansion to find alternative word endings (e.g., autis *=autism, autistic, etc.).

## Supplementary Materials

The following supporting information can be downloaded at: https://www.mdpi.com/article/10.3390/brainsci12050602/s1. Table S1: A summarization of the articles included in the review, including levels of evidence, participant characteristics, study design, and methods.
