# Supplementary material for: Neuroimaging Techniques as Descriptive and Diagnostic Tools for Infants at Risk for Autism Spectrum Disorder: A Systematic Review
Source: Brain Sci. 2022 May 5;12(5):602. doi: 10.3390/brainsci12050602 (PMC9139416; doi:10.3390/brainsci12050602)
Supplement: Supplementary file 1 [file brainsci-12-00602-s001.zip › brainsci-1671724-supplementary.pdf]

Table S1: Results

Authors, year	Title	Journal	Study design & Sample Size	Population	Age range	Types of Neuroimaging Used	Category
**Bhat et al., 2019**	Exploring cortical activation and connectivity in infants with and without familial risk for autism during naturalistic interactions: A preliminary study	Infant Behavior and Development	Cross-sectional; N = 15	ASD-HR (n = 9) and ASD-LR (n = 6) infants	6 - 9 months	fNIRS	Descriptive
**Blasi et al., 2015**	Atypical processing of voice sounds in infants at risk for autism spectrum disorder	Cortex	Cross-sectional; N = 33	ASD-HR (n = 15) and ASD-LR (n = 18) infants	4 - 7 months	fMRI	Descriptive
**Braukamann et al., 2018**	Diminished socially selective neural processing in 5-month-old infants at high familial risk of autism	European Journal of Neuroscience	Cross-sectional; N = 29	ASD-HR (n = 16) and ASD-LR (n = 13) infants	5 months	fNIRS	Descriptive
**Cardenas-de-la-Parra et al., 2021**	A voxel-wise assessment of growth differences in infants developing autism spectrum disorder	Neuroimage: Clinical	Longitudinal; N = 503	ASD-HR+ (n = 56), ASD-HR- (n = 285), and ASD-LR (n = 162) infants	6, 12, & 24 months	MRI	Diagnostic
**Damiano-Goodwin et al., 2017**	Developmental sequelae and neurophysiologic substrates of sensory seeking in infant siblings of children with autism spectrum disorder	Developmental Cognitive Neuroscience	Longitudinal; N = 40	ASD-HR+ (n = 6), ASD-HR- (n = 14), and ASD-LR (n = 20) infants	18 & 36 months	EEG	Diagnostic
**Darki et al., 2021**	T1-Weighted/T2-Weighted ratio mapping at 5 months captures individual differences in behavioral development and differentiates infants at familial risk for autism from controls	Cerebral Cortex	Cross-sectional; N = 46	ASD-HR (n = 29) and ASD-LR (n = 17) infants	~5 months	MRI	Descriptive
**Dickinson et al., 2021**	Multivariate neural connectivity patterns in early infancy predict later autism symptoms	Society of Biological Psychiatry	Longitudinal; N = 65	ASD-HR (n = 36) and ASD-LR (n = 29) infants	3 & 18 months	EEG	Diagnostic

**Edwards et al., 2017**	Differences in neural correlates of speech perception in 3 month olds at high and low risk for autism spectrum disorder	Journal of Autism and Developmental Disorders	Cross-sectional; N = 38	ASD-HR (n = 21) and ASD-LR (n = 17) infants	3 months	fNIRS	Descriptive
**Elison et al., 2013**	White matter microstructure and atypical visual orienting in 7- month olds at risk for autism	American Journal of Psychiatry	Longitudinal; N = 97	ASD-HR+ (n = 16), ASD-HR- (n = 40), and ASD-LR (n = 41) infants	7 & 25 months	DTI	Diagnostic
**Elsabbagh et al., 2015**	Infant neural sensitivity to dynamic gaze relates to quality of parent-infant interaction at 6-8 months in infant at risk for autism	Journal of Autism and Developmental Disorders	Cross-sectional; N = 92	ASD-HR (n = 45) and ASD-LR (n = 47) infants	7 months	EEG	Descriptive
**Finch et al., 2017**	Lateralization of ERPs to speech stimuli and handedness in the early developmental of autism spectrum disorder.	Journal of Neurodevelopmental Disorders	Longitudinal; N = 163	ASD-HR+ (n = 23), ASD-HR- (n = 67), and ASD-LR (n = 73) infants	EEG at 12 months; behavioral assessment at 36 months	EEG	Diagnostic
**Finch, Tager-Flusberg, & Nelson, 2018**	Neural responses to linguistic stimuli in children with and without autism spectrum disorder	European Journal of Neuroscience	Cross-sectional; N = 134 (85 contributed usable EEG)	ASD-HR+ (n = 14), ASD-HR- (n = 29), and ASD-LR (n = 42) infants	36 months	EEG	Diagnostic
**Gabard-Durnam et al., 2015**	Alpha asymmetry in infants at risk for autism spectrum disorders	Journal of Autism and Developmental Disorders	Longitudinal; N = 108	ASD-HR (n = 57) and ASD-LR (n = 51) infants	6, 12, & 18 months	EEG	Descriptive
**Guy et al., 2017**	Neural correlates of face processing in etiologically-distinct 12-month-old infants at high-risk of autism spectrum disorder	Developmental Cognitive Neuroscience	Cross-sectional; N = 57	ASD-LR (n = 21), ASD-HR (n = 21), and infants with Fragile X Syndrome (n = 15)	12 months	EEG	Descriptive
**Haarsten et al., 2019**	Functional EEG connectivity in infants associates with later restricted and repetitive behaviours in autism; a replication study	Translational Psychiatry	Longitudinal; N = 101	ASD-HR+ (n = 13), ASD-HR- (n = 47), ASD-HR showing atypical development (n = 21), and ASD-LR (n = 47) infants	14 months old (13 - 18 months)	EEG	Diagnostic

**Hazlett et al., 2012**	Brain volume findings in 6-month-old infants at high familial risk for autism	American Journal of Psychiatry	Cross-sectional; N = 134	ASD-HR (n = 98) and ASD-LR (n = 36) infants	6 months	MRI	Descriptive
**Hazlett et al., 2017**	Early brain development in infants at high risk for autism spectrum disorder	Nature	Longitudinal; N = 435	ASD-HR+ (n = 70), ASD-HR- (n = 248), and ASD-LR (n = 117) infants	6, 12, & 24 months	MRI	Diagnostic
**Jones et al., 2016**	Reduced engagement with social stimuli in 6-month-old infants with later autism spectrum disorder: A longitudinal prospective study of infants at high familial risk	Journal of Neurodevelopmental Disorders	Longitudinal; N = 88	ASD-HR (n = 43) and ASD-LR (n = 45) infants	6, 12, 18, & 24 months	EEG	Diagnostic
**Jones et al., 2017**	Parent-delivered early intervention in infants at risk for autism spectrum disorder: Effects on electrophysiological and habituation measures of social attention	Autism Research	Longitudinal; N = 33	ASD-HR infants (n = 33)	EEG at 6, 12, & 18 months; intervention between 9 - 11 months	EEG	Descriptive
**Keehn et al., 2013**	Functional connectivity in the first year of life in infants at-risk for autism: A preliminary near-infrared spectroscopy study	Frontiers in Human Neuroscience	Longitudinal; N = 64	ASD-HR (n = 27) and ASD-LR (n = 37) infants	3,6,9, & 12-months	fNIRS	Descriptive
**Keehn et al., 2015**	Atypical hemispheric specialization for faces in infants-at-risk for autism spectrum disorder	Autism Research	Longitudinal; N = 95 (60 with longitudinal data/diagnostic outcomes)	ASD-HR+ (n = 10), ASD-HR- (n = 24), and ASD-LR (n = 26) infants	6 & 12 months	EEG	Diagnostic
**Key & Stone, 2012**	Processing of novel and familiar faces in infants at average and high risk for autism	Developmental Cognitive Neuroscience	Cross-sectional; N = 35	ASD-HR (n = 15) and ASD-LR (n = 20) infants	9 months	EEG	Descriptive
**Key & Stone, 2012**	Same but different: Nine-month-old infants at low and high risk for autism look at the same facial features but process them	Autism Research	Cross-sectional; N = 35	ASD-HR (n = 15) and ASD-LR (n = 20) infants	9 months	EEG	Descriptive

	using different brain mechanisms						
**Key et al., 2015**	Positive affect processing and joint attention in infants at high risk for autism: An exploratory study	Journal of Autism and Developmental Disorders	Longitudinal; N = 31	ASD-HR (n = 16) and ASD-LR (n = 15) infants	9-15 months	EEG	Descriptive
**Kolesnik et al., 2019**	Increased cortical reactivity to repeated tones at 8 months in infants with later autism spectrum disorder	Translational Psychiatry	Longitudinal; N = 143	ASD-HR (n = 116; 14 of which were later ASD-HR+) and ASD-LR (n = 27) infants	8 & 14 months, 2 & 3 years	EEG	Diagnostic
**Levin et al., 2017**	EEG power at 3 months in infants at high familial risk for autism	Journal of Neurodevelopmental Disorders	Longitudinal; N = 48	ASD-HR (n = 29; 25 with sufficient EEG data, 7 of which were later ASD-HR+) and ASD-LR (n = 19) infants	3, 6, 9, 12, 18, 24, & 36 months	EEG	Diagnostic
**Lewis et al., 2017**	The emergence of network inefficiencies in infants with autism spectrum disorder	Biological Psychiatry	Longitudinal; N = 260 (116 with longitudinal data)	ASD-HR+ (n = 15), ASD-HR- (n = 66), and ASD-LR (n = 35) infants	6 & 12 months	MRI	Diagnostic
**Liu et al., 2020**	Emerging atypicalities in functional connectivity of language-related networks in young infants at high familial risk for ASD	Developmental Cognitive Neuroscience	Longitudinal; N = 65	ASD-HR (n = 33) and ASD-LR (n = 32) infants	1.5 - 9 months	fMRI	Descriptive
**Liu et al., 2019**	Altered lateralization of dorsal language tracts in 6-week-old infants at risk for autism	Developmental Science	Longitudinal; N = 34	ASD-HR (n = 19) and ASD-LR (n = 15) infants	6 weeks, 18 months, & 36 months	DTI	Diagnostic
**Lloyd-Fox et al., 2013**	Reduced neural sensitivity to social stimuli in infants at risk for autism	Proceedings of the Royal Society B	Cross-sectional; N = 34	ASD-HR (n=18) and ASD-LR (n=16) infants	4 – 6 months	fNIRS	Descriptive
**Llyod-Fox et al., 2018**	Cortical responses before 6 months of life associate with later autism	European Journal of Neuroscience	Longitudinal; N = 36	ASD-HR+ (n = 5), ASD-HR- (n = 15), and ASD-LR (n = 16) infants	4 - 6 months; follow-up at 36 months	fNIRS	Diagnostic
**Luyster et al., 2011**	Neural correlates of familiar and unfamiliar face	Brain Topography	Cross-sectional; N = 56	ASD-HR (n = 32) and ASD-LR (n =24) infants	12 months	EEG	Descriptive

	processing in infants at risk for autism						
**Luyster et al., 2014**	Neural measures of social attention across the first years of life: Characterizing typical development and markers of autism risk	Developmental Cognitive Neuroscience	Longitudinal; N = 260	ASD-HR (n = 123) and ASD-LR (n = 137) infants	6 - 36 months	EEG	Descriptive
**MacDuffie et al., 2020**	Sleep onset problems and subcortical development in infants later diagnosed with autism spectrum disorder	American Journal of Psychiatry	Longitudinal; N = 432	ASD-HR+ (n = 71), ASD-HR- (n = 234), and ASD-LR (n = 127) infants	6, 12, & 24 months	MRI	Diagnostic
**McCleery et al., 2009**	Atypical face versus object processing and hemispheric asymmetries in 10-month-old infants at risk for autism	Biological Psychiatry	Cross-sectional; N = 40	ASD-HR (n = 20) and ASD-LR (n = 20) infants	10 months	EEG	Descriptive
**McKinnon et al., 2019**	Restricted and repetitive behavior and brain functional connectivity in infants at risk for developing autism spectrum disorder	Biological Psychiatry	Longitudinal; N = 38	Infants; some meeting criteria for ASD diagnosis (n = 20) and some not meeting the criteria (n = 18)	12 & 24 months	fMRI	Diagnostic
**Nair et al., 2021**	Altered thalamocortical connectivity in 6-week-old infants at high familial risk for autism spectrum disorder	Cerebral Cortex	Longitudinal; N = 52	ASD-HR (n = 24) and ASD-LR (n = 28) infants	6 weeks; later follow-up	Functional connectivity MRI, DTI	Diagnostic
**Orekhova et al., 2014**	EEG hyper-connectivity in high-risk infant is associated with later autism	Journal of Neurodevelopmental Disorders	Longitudinal; N = 54	ASD-HR+ (n = 10), ASD-HR- (n = 18), and ASD-LR (n = 28) infants	EEG at 14 months; ASD at 36 months	EEG	Diagnostic
**Pecukonis et al., 2021**	Exploring the relation between brain response to speech at 6-months and language outcomes at 24-months in infants at high and ASD-LR: A preliminary functional near-infrared spectroscopy study	Developmental Cognitive Neuroscience	Longitudinal; N = 32	ASD-HR+ (n = 5), ASD-HR- (n = 9), and ASD-LR (n = 18) infants	6 & 24 months	fNIRS	Diagnostic

**Peterson et al., 2021**	Evidence for normal extra-axial cerebrospinal fluid volume in autistic males from middle childhood to adulthood	Neuroimage	Longitudinal; N = 189	Autistic (n = 92) and non-autistic (n = 97) males	3 - 42 years	MRI	Descriptive
**Pote et al., 2019**	Familial risk of autism alters subcortical and cerebellar brain anatomy in infants and predicts the emergence of repetitive behaviors in early childhood	Autism Research	Longitudinal; N = 50	ASD-HR (n = 24) and ASD-LR (n = 26) infants	4 - 6 months & 36 months	MRI	Diagnostic
**Righi et al., 2014**	Functional connectivity in the first year of life in infants at risk for autism spectrum disorder: An EEG study	PLOS One	Longitudinal; N = 54	ASD-HR (n = 28) and ASD-LR (n = 26) infants	6 - 12 months	EEG	Diagnostic
**Schumann et al., 2010**	Longitudinal magnetic resonance imaging study of cortical development through early childhood in autism	The Journal of Neuroscience	Longitudinal; N = 85	Toddlers with ASD symptoms (n = 41) and without ASD symptoms (n = 44)	1.5 - 5 years	MRI	Descriptive
**Seery et al., 2013**	Atypical lateralization of ERP response to native and non-native speech in infants at risk for autism spectrum disorder	Developmental Cognitive Neuroscience	Longitudinal; N = 108	ASD-HR (n = 62) and ASD-LR (n = 46) infants	6, 9, & 12 months	EEG	Descriptive
**Seery, Tager-Flusberg, & Nelson, 2014**	Event-related potentials to repeated speech in 9-month-old infants at risk for autism spectrum disorder	Journal of Neurodevelopmental Disorders	Cross-sectional; N = 80	ASD-HR (n = 35) and ASD-LR (n = 45) infants	9 months	EEG	Descriptive
**Shen et al., 2013**	Early brain enlargement and elevated extra-axial fluid in infants who develop autism spectrum disorder	Brain	Longitudinal; N = 55	ASD-HR (n = 33; 10 of which were later ASD-HR+) and ASD-LR (n= 22) infants	6 - 9 months, 12 - 15 months, & 18 - 24 months	MRI	Diagnostic
**Shen et al., 2017**	Increased extra-axial cerebrospinal fluid in high-risk infants who later develop autism	Biological Psychiatry	Longitudinal; N = 343	ASD-HR+ (n = 47), ASD-HR- (n = 174), and ASD-LR (n = 122) infants	6, 12, & 24 months	MRI	Diagnostic

**Simon et al., 2017**	Neural correlates of sensory hyporesponsiveness in toddlers at high risk for autism spectrum disorder	Journal of Autism and Developmental Disorders	Cross-sectional; N = 22	ASD-HR infants (n = 22)	18 months	EEG	Descriptive
**Swanson et al., 2017**	Subcortical brain and behavior phenotypes differentiate infants with autism versus language delay	Biological Psychiatry	Longitudinal; N = 525	ASD-HR+ (n = 86), ASD-HR- (n = 255), ASD-HR with language delay (n = 41), and ASD-LR (n = 143) infants	6, 12, & 24 months	MRI	Diagnostic
**Tierney et al., 2012**	Developmental trajectories of resting EEG power: An endophenotype of autism spectrum disorder	PLOS One	Longitudinal; N = 122	ASD-HR (n = 65) and ASD-LR (n = 57) infants	6, 9, 12, 18, & 24 months	EEG	Descriptive
**Wilkinson et al., 2019**	Reduced frontal gamma power at 24 months is associated with better expressive language in toddlers at risk for autism	Autism Research	Cross sectional; N = 101	ASD HR+ (n = 16), ASD-HR- (n = 42), and ASD-LR (n = 43) infants	EEG at 24 months; ASD assessment between 24-36 months	EEG	Diagnostic
**Wilkinson et al., 2019**	Use of longitudinal EEG measures in estimating language development in infants with and without familial risk for autism spectrum disorder	Neurobiology of Language	Longitudinal; N = 130	ASD-HR+ (n = 21), ASD-HR- (n = 51), and ASD-LR (n = 58) infants	3 - 24 months	EEG	Diagnostic
**Wolff et al. 2012**	Differences in white matter fiber tract development present from 6 to 24 months in infants with autism	American Journal of Psychiatry	Longitudinal; N = 92	ASD-HR+ (n = 28) and ASD-LR (n = 64) infants	6, 12, & 24 months	DTI	Diagnostic
**Wolff et al., 2015**	Altered corpus callosum morphology associated with autism over the first 2 years of life	Brain	Longitudinal; N = 378	ASD-HR+ (n = 57), ASD-HR- (n = 213), and ASD-LR (n = 108) infants	6, 12, & 24 months	DTI & MRI	Diagnostic

Table Key:

**Abbreviation**:	**Definition**:
ASD	Autism Spectrum Disorder
ASD-HR	High Risk for ASD (due to familial diagnosis)
ASD-HR+	High Risk for ASD; ASD Diagnosis Confirmed
ASD-HR-	High Risk for ASD; NoASD Diagnosis Received
ASD-LR	Low Risk for ASD
fMRI	Functional Magnetic Resonance Imaging
MRI	Magnetic Resonance Imaging
EEG	Electroencephalography
fNIRS	Functional Near-Infrared Spectroscopy
DTI	Diffusion Tensor Imaging
